# Cerebrospinal fluid (CSF) augments metabolism and virulence expression factors in *Acinetobacter baumannii*

**DOI:** 10.1038/s41598-021-81714-6

**Published:** 2021-02-26

**Authors:** Jasmine Martinez, Chelsea Razo-Gutierrez, Casin Le, Robert Courville, Camila Pimentel, Christine Liu, Sammie E. Fung, Marisel R. Tuttobene, Kimberly Phan, Alejandro J. Vila, Parvin Shahrestani, Veronica Jimenez, Marcelo E. Tolmasky, Scott A. Becka, Krisztina M. Papp-Wallace, Robert A. Bonomo, Alfonso Soler-Bistue, Rodrigo Sieira, Maria Soledad Ramirez

**Affiliations:** 1 Center for Applied Biotechnology Studies, Department of Biological Science, College of Natural Sciences and Mathematics, California State University Fullerton, 800 N State College Blvd, Fullerton, CA 92831 USA; 2 Instituto de Biología Molecular Y Celular de Rosario (IBR, CONICET-UNR), Rosario, Argentina; 3 Área Biofísica, Facultad de Ciencias Bioquímicas Y Farmacéuticas, Universidad Nacional de Rosario, Rosario, Argentina; 4 Research Service and GRECC, Department of Veterans Affairs Medical Center, Louis Stokes Cleveland, Cleveland, OH USA; 5 Departments of Medicine, Pharmacology, Molecular Biology and Microbiology, Biochemistry, Proteomics and Bioinformatics, Case Western Reserve University School of Medicine, Cleveland, OH USA; 6 CWRU-Cleveland VAMC Center for Antimicrobial Resistance and Epidemiology (Case VA CARES), Cleveland, OH USA; 7 Instituto de Investigaciones Biotecnológicas, Universidad Nacional de San Martín-Consejo Nacional de Investigaciones Científicas Y Técnicas, San Martín, Buenos Aires, Argentina; 8 Fundación Instituto Leloir – IIBBA CONICET, Buenos Aires, Argentina

**Keywords:** Microbiology, Pathogenesis

## Abstract

In a recent report by the Centers for Disease Control and Prevention (CDC), multidrug resistant (MDR) *Acinetobacter baumannii* is a pathogen described as an “urgent threat.” Infection with this bacterium manifests as different diseases such as community and nosocomial pneumonia, bloodstream infections, endocarditis, infections of the urinary tract, wound infections, burn infections, skin and soft tissue infections, and meningitis. In particular, nosocomial meningitis, an unwelcome complication of neurosurgery caused by extensively-drug resistant (XDR) *A. baumannii*, is extremely challenging to manage. Therefore, understanding how *A. baumannii* adapts to different host environments, such as cerebrospinal fluid (CSF) that may trigger changes in expression of virulence factors that are associated with the successful establishment and progress of this infection is necessary. The present in-vitro work describes, the genetic changes that occur during *A. baumannii* infiltration into CSF and displays *A. baumannii*’s expansive versatility to persist in a nutrient limited environment while enhancing several virulence factors to survive and persist. While a hypervirulent *A. baumannii* strain did not show changes in its transcriptome when incubated in the presence of CSF, a low-virulence isolate showed significant differences in gene expression and phenotypic traits. Exposure to 4% CSF caused increased expression of virulence factors such as fimbriae, pilins, and iron chelators, and other virulence determinants that was confirmed in various model systems. Furthermore, although CSF's presence did not enhance bacterial growth, an increase of expression of genes encoding transcription, translation, and the ATP synthesis machinery was observed. This work also explores *A. baumannii’s* response to an essential component, human serum albumin (HSA), within CSF to trigger the differential expression of genes associated with its pathoadaptibility in this environment.

## Introduction

*Acinetobacter baumannii* has emerged as an important pathogen due to its ability to resist multiple antibiotics, persist in hospital settings, and cause a wide variety of infections such as pneumonia, bacteremia, urinary tract infections, skin and soft-tissue infections, and meningitis, showing its capacity to infect diverse host environments (lung, blood, CSF, etc.)^1–4^. The acquisition of resistance to carbapenems by certain strains (carbapenem-resistant *Acinetobacter baumannii*, CRAB) increased the problematic nature of this pathogen^5,6^, which has been qualified as an “urgent” threat in a recent report by the Centers for Disease Control and Prevention^7^.

Bacterial meningitis, which is considered a medical emergency, is a serious infection that can cause permanent disabilities (brain damage, hearing loss, and learning problems) or death if untreated^8–10^. Post neurosurgical *A. baumannii* meningitis is reported between ~ 10–17% of hospitalized inpatients^11–13^, can cause death or leave permanent sequelae, and is usually associated with high mortality rates reaching up to 40 to 70%^14,15^. Illustrative of these infections' dangerous nature is the recent reported case of the *A. baumannii* infection of a 39-year-old man treated with external ventricular drainage of cerebrospinal fluid (CSF)^16^. Although the strain was susceptible to colistin at the time of detection, it quickly acquired resistance while maintaining virulence^16^. This genetic plasticity, a consequence of its ability to acquire and integrate foreign DNA, gives *A. baumannii* a tremendous metabolic versatility that permits the bacterium to adapt and persist in harsh conditions^2,17–21^. *A. baumannii's* success in causing numerous infections, where it gets in contact with different body components and fluids, must be the result of its capabilities to not only capture adequate genetic determinants, but also regulate expression of the proper cell components^2,6,17–19,22–24^. Previously, we demonstrated that human serum albumin (HSA) and pleural fluid (HSA-containing fluid) affect *A. baumannii* behavior, triggering an adaptive response that modulates DNA uptake, cytotoxicity, immune evasion, stress responses and metabolism^22,24–26^.

Understanding the virulence determinants of this bacterium requires a thorough comprehension of the general genotypic and phenotypic responses when it is exposed to different human body fluids. As part of our studies on *A. baumannii* pathogenicity in relation to meningitis, we assessed in these experiments how gene expression changes when in contact with CSF. Our goal was to enhance our understanding of why this disease and pathogen are so problematic with the intent to develop better therapies for this fatal infection.

## Results and discussion

### CSF enhances the expression of genes involved in transcription and translation machineries, ATP production, and specific metabolic pathways without increasing growth rate

To uncover the impact of specific host environments at the transcriptional level, two different *A. baumannii* strains, A118 (low pathogenicity and high antibiotic susceptibility) and AB5075 (hypervirulent and multi-drug resistant), were exposed to CSF^27–29^. Transcriptomic analysis of *A. baumannii* A118, using a fold-change cutoff of log_2_ > 1 (with adjusted *P*-value < 0.05), showed 275 differentially expressed-genes (DEGs), 7.76% of the total genes in the *A. baumannii* A118 reference genome. However, statistically significant changes were not observed when *A. baumannii* AB5075, a hypervirulent and highly resistant strain, was exposed to CSF under the conditions tested. Previous observations have shown that *A. baumannii’s* response to different stimuli correlates with each particular strain’s degree of pathogenicity. Less pathogenic strains induced more changes in their phenotypic behavior to overcome the stressful environment and persist^30^.

The analysis of *A. baumannii* A118 DEGs revealed an increase in the expression of many genes involved in gene expression processes and energy production machineries (Table 1 and Supplementary Table S1). Notably, a large proportion of ribosomal protein genes are overexpressed upon exposure to CSF. Among the ribosomal protein associated genes, 47 out of 55 displayed a significant increase of expression of twofold or more. Coincidently, key translation genes such as those encoding elongation factors (EF) EF-G, EF-F and EF-P were also up-regulated. Concurrently, the main genes of the transcriptional machinery (RNA polymerase) were similarly overexpressed. The *rpoB* and *rpoC* genes, which code for the beta and beta' subunits of RNA polymerase (core of the transcription machinery), were overexpressed with a log_2_fold just below 1. However, the gene encoding the alpha subunit was also upregulated with a log_2_fold change of 1.48 (Table 1 and Supplementary Table S1).

**Table 1:** CSF regulated genes in *A. baumannii* A118.

Gene ID	Gene name	Log_2_fold change	p-adj	Gene associated function
AbA118F_3259	*bauB*	3.55	1.4 E-21	Iron compound ABC uptake transporter substrate-binding protein
AbA118F_1017	*fimA*	3.09	1.2 E-160	Fimbrial protein
AbA118F_3260	*bauE*	2.84	1.1 E-06	putative iron compound ABC uptake transporter, ATP-binding protein
AbA118F_0516		2.81	3.4 E-126	Exporter protein, RND family
AbA118F_0517		2.79	9.5 E-142	Polyketide synthase module
AbA118F_0746		2.77	9.1 E-36	hypothetical protein
AbA118F_0519		2.58	2.2 E-94	Acyl-CoA dehydrogenase
AbA118F_0523		2.54	1.1 E-63	Autoinducer synthesis protein SolI
AbA118F_3262	*bauD*	2.52	2.9 E-02	Iron transport protein
AbA118F_0514		2.50	1.2 E-117	hypothetical protein
AbA118F_0518		2.45	2.2 E-66	Acyl carrier protein
AbA118F_0515		2.39	2.4 E-65	hypothetical protein
AbA118F_0745		2.37	1.9 E-12	Hypothetical protein
AbA118F_2656		2.34	9.0 E-3	Urease beta subunit
AbA118F_3134	*fimB*	2.32	4.7 E-18	P pilus assembly protein, chaperone PapD
AbA118F_1015		2.30	1.1 E-124	outer membrane usher protein precursor
AbA118F_2504		2.16	1.9 E-102	Biotin carboxylase of acetyl-CoA carboxylase
AbA118F_0483		2.14	1.2 E-107	ATP synthase delta chain
AbA118F_1447		2.12	6.7 E-08	hypothetical protein
AbA118F_2933	*rpmC*	2.12	4.5 E-85	LSU ribosomal protein L29p (L35e)-*rpmC*
AbA118F_1014		2.10	1.5 E-78	fimbrial adhesin precursor
AbA118F_2505		2.09	1.4 E-58	Biotin carboxyl carrier protein of acetyl-CoA carboxylase
AbA118F_3136		2.09	8.8 E-19	Fimbrial adhesin
AbA118F_0481		2.08	7.4 E-108	ATP synthase gamma chain
AbA118F_2932		2.08	3.0 E-94	SSU ribosomal protein S17p (S11e)
AbA118F_0041		2.03	1.2 E-90	33–36 kDa outer membrane protein
AbA118F_2934		1.99	1.3 E-92	LSU ribosomal protein L16p (L10e)
AbA118F_3202		1.97	1.3 E-96	Translation elongation factor Ts
AbA118F_1819		1.97	1.3 E-104	LSU ribosomal protein L10p (P0)
AbA118F_2936		1.96	1.7 E-92	LSU ribosomal protein L22p (L17e)
AbA118F_2935		1.96	2.1 E-93	SSU ribosomal protein S3p (S3e)
AbA118F_1818		1.93	5.6 E-95	LSU ribosomal protein L7p/L12p (P1/P2)
AbA118F_0484	*atpF*	1.92	2.7 E-90	ATP synthase F0 sector subunit b
AbA118F_0113		1.91	7.9 E-58	hypothetical protein
AbA118F_3133		1.91	9.3 E-43	Fimbrial protein precursor
AbA118F_2937		1.90	1.5 E-88	SSU ribosomal protein S19p (S15e)
AbA118F_3135		1.89	3.5 E-08	type 1 fimbriae anchoring protein FimD
AbA118F_0482	*atpA*	1.88	7.0 E-97	ATP synthase alpha chain
AbA118F_2359		1.87	1.5 E-05	hypothetical protein
AbA118F_3256	*basD*	1.86	2.1 E-08	Non-ribosomal peptide synthetase modules, siderophore biosynthesis
AbA118F_1925		1.85	4.2 E-55	Succinyl-CoA ligase [ADP-forming] beta chain
AbA118F_0480	*atpD*	1.83	9.1 E-76	ATP synthase beta chain
AbA118F_1016		1.80	2.1 E-67	P pilus assembly protein, chaperone PapD
AbA118F_2749		1.77	1.5 E-08	Homocysteine S-methyltransferase-like protein
AbA118F_0513		1.76	4.2 E-11	4′-phosphopantetheinyl transferase
AbA118F_3345		1.76	1.8 E-20	Hypothetical protein
AbA118F_1919		1.76	2.5 E-14	tRNA-Thr
AbA118F_3265	*basB*	1.76	3.8 E-08	Non-ribosomal peptide synthetase
AbA118F_2938		1.75	2.9 E-79	LSU ribosomal protein L2p (L8e)
AbA118F_2939		1.75	1.1 E-73	LSU ribosomal protein L23p (L23Ae)
AbA118F_0485		1.73	3.2 E-66	ATP synthase F0 sector subunit c
AbA118F_2311		1,72	2.3 E-49	SSU ribosomal protein S18p : SSU ribosomal protein S18p, zinc-independent
AbA118F_0479	*atpC*	1.72	3.8 E-64	ATP synthase epsilon chain
AbA118F_3254	*basF/entF*	1.72	1.6 E-04	Isochorismatase of siderophore biosynthesis
AbA118F_2312		1.71	1.7 E-66	SSU ribosomal protein S6p
AbA118F_1530		1.69	2.0 E-30	hypothetical protein
AbA118F_3044		1.68	5.0 E-62	3-oxoacyl-[acyl-carrier protein] reductase
AbA118F_0520		1.68	3.7 E-41	Polyketide synthase module
AbA118F_2927		1.67	6.2 E-71	SSU ribosomal protein S8p (S15Ae)
AbA118F_3258	*bauA*	1.67	1.9 E-11	Ferrichrome-iron receptor
AbA118F_3481		1.67	3.4 E-14	Oxidoreductase, short-chain dehydrogenase/reductase family
AbA118F_3201		1.66	2.9 E-02	hypothetical protein
AbA118F_1820		1.66	9.5E-72	LSU ribosomal protein L1p (L10Ae)
AbA118F_1821		1.64	2.3 E-69	LSU ribosomal protein L11p (L12e)
AbA118F_1340		1.64	1.5 E-30	Ferrichrome-iron receptor
AbA118F_0638	*tssL*	1,63	1.2 E-39	Putative transmembrane protein
AbA118F_2322	*pgaB*	1.63	1.5 E-19	Biofilm PGA synthesis deacetylase PgaB
AbA118F_2321	*pgaA*	1.63	2.1 E-26	Biofilm PGA outer membrane secretin PgaA
AbA118F_0387		1.61	1.3 E-54	Hemolysin
AbA118F_2915		1.60	2.9 E-53	LSU ribosomal protein L17p
AbA118F_2024		1.59	9.3 E-43	Sodium-alanine symporter family protein
AbA118F_2923		1.59	4.0 E-42	LSU ribosomal protein L30p (L7e)
AbA118F_1509		1.59	1.8 E-63	Translation elongation factor Tu
AbA118F_0149		1.58	8.3 E-26	Imidazolonepropionase
AbA118F_2323	*pgaC*	1.56	2.8 E-13	Biofilm PGA synthesis N-glycosyltransferase PgaC
AbA118F_2920		1.56	6.5 E-44	LSU ribosomal protein L36p : LSU ribosomal protein L36p, zinc-dependent
AbA118F_1621		1.55	4.6 E-54	Glyceraldehyde-3-phosphate dehydrogenase, putative
AbA118F_1623		1.55	2.0 E-43	Gluconate transporter family protein
AbA118F_3243		1.54	3.1 E-52	hypothetical protein
AbA118F_0791		1.54	1.0 E-50	Phosphoglycerate kinase
AbA118F_2940		1.53	8.5 E-60	LSU ribosomal protein L4p (L1e)
AbA118F_2921		1.53	4.0 E-65	Protein translocase subunit SecY
AbA118F_3255	*entE/basE*	1.52	2.5 E-06	2,3-dihydroxybenzoate-AMP ligase of siderophore biosynthesis
AbA118F_0114		1.52	5.7 E-29	hypothetical protein
AbA118F_0148		1.51	2.6 E-11	Formiminoglutamase
AbA118F_2924		1.51	1.3 E-48	SSU ribosomal protein S5p (S2e)
AbA118F_1954		1.50	7.5 E-53	LSU ribosomal protein L21p
AbA118F_3316		-1.50	1.59 E-29	Phosphate ABC transporter, periplasmic phosphate-binding protein PstS
AbA118F_1136		-1.52	2.8 E-04	putative zinc-type alcohol dehydrogenase-like protein YbdR
AbA118F_1643		-1.56	5.3 E-04	Twin-arginine translocation protein TatB
AbA118F_0751		-1.57	1.6 E-04	TPR-repeat-containing protein
AbA118F_2679		-1.69	1.5 E-02	hypothetical protein
AbA118F_1644		-1.82	5.8 E-05	Twin-arginine translocation protein TatC
AbA118F_2820		-1.86	3.9 E-02	Allantoin racemase
AbA118F_2477		-1.87	4.6 E-10	Mg(2 +)-transport-ATPase-associated protein MgtC
AbA118F_0711		-1.89	5.4 E-16	Acyl-CoA dehydrogenase
AbA118F_0704		-1.99	1.4 E-02	2-aminoethylphosphonate ABC transporter substrate-binding protein
AbA118F_2199		-2.06	2.4 E-04	Protein co-occuring with molybdenum cofactor biosynthesis protein B
AbA118F_0907		-2.14	5.2 E-20	outer membrane porin, putative
AbA118F_2367		-2.30	4.0 E-02	Phage tail/DNA circulation protein
AbA118F_1645		-2.39	1.2 E-27	Alkaline phosphatase
AbA118F_1190		-2.62	4.9 E-02	Lysozyme (N-acetylmuramidase) family
AbA118F_1376		-2.93	3.4 E-02	Lysozyme (N-acetylmuramidase) family
AbA118F_3137		-3.07	2.8 E-03	hypothetical protein
AbA118F_2475		-3.31	2.0 E-08	hypothetical protein
AbA118F_2476		-3.83	2.7 E-11	Mg(2 +) transport ATPase, P-type

The table lists all *A. baumanii* A118 ORFs that are regulated by CSF with a Log2FC ≥|1.5|, p < 0.05.

In addition, genes important for energy production in the cell were also upregulated upon CSF exposure. The *atpIBEFHAGCD* locus, an operon encoding the FoF_1_-ATP synthase (the main ATP generator in the bacterial cell) displayed a threefold transcriptional increase in expression (Table 1 and Supplementary Table S1). These transcriptional responses suggest that when CSF is present in the environment, there is an increase in expression of transcription- and translation-related genes, as well as of FoF1-ATP synthase, the main ATP generator. Also, CSF exposure induces the transcription of specific metabolic routes in *A. baumannii*. In particular, several dehydrogenases of the tricarboxylic acid cycle intermediates as well as a citrate symporter were significantly overexpressed, together with two proline symporters (Supplementary Table S1). The transcriptomic data showed that type I glutamine synthetase (AbA118F_3228) and the proline symporter *putP* genes are upregulated by a log_2_fold change of 0.58 and 0.87 respectively.

Studies on *Salmonella typhimurium* showed that *putP* codes for a proline permease, an integral membrane protein, that is the primary transport protein when this amino acid is the only carbon or nitrogen source^31^. The transcriptomic analysis showed upregulation (log_2_fold change 0.62) of AbA118F_2664, a gene that encodes a CitMHS citrate-magnesium hydrogen complex symporter (Supplementary Table S1). Proteins of the CitMHS family transport citrate-Mg^2+^ complex coupled with one proton per complex molecule^32^. Interestingly, increased citrate levels help survival of *A. baumannii* in certain conditions^26^. Thus, the net effect of CSF might be an increase in expression of this transporter, which would lead to higher citrate intracellular concentrations that may result in increased rate of growth.

While most of the DEGs were upregulated, only 66 showed a lower transcription level. Among the most downregulation genes we found those coding for transporters (e.g. Mg2 + transporters, AbA118F_2476 and AbA118F_2477) and catabolic proteins (such as AbA118F_1645 Alkaline Phosphatase and AbA118F_0711 Acyl-CoA dehydrogenase) (Table 1 and Supplementary Table S1).

Gene ontology (GO) analysis was next undertaken to identify molecular functions and biological pathways associated to *A*. *baumannii’*s adaptive responses to CSF. Consistent with the above mentioned DEGs, GO enrichment analysis revealed a statistically significant overrepresentation of the GO categories ATP synthesis coupled proton transport, translation, tricarboxylic acid cycle, and aerobic respiration by 13.8-, 8.8-, 4.9- and 4.3-fold, respectively (adj. *P*-value < 0.05). Other studies found that exposure of *A. baumannii* strains to amikacin, imipenem, and meropenem was associated with increased expression of genes involved in the tricarboxylic acid cycle, biosynthesis of amino acids, purines, and pyrimidines, as well as the operons related to ATP, RNA, and protein synthesis^33^. Taken together, these results suggest that various stressful environments (low nutrient availability and antibiotic treatment) induce expression of genes associated to energy production, protein synthesis, and metabolism in *A. baumannii*. As a consequence of these transcriptomic changes, the bacterial cell adapts to survive and accelerate metabolism rive in these hostile conditions.

**Figure 1:**
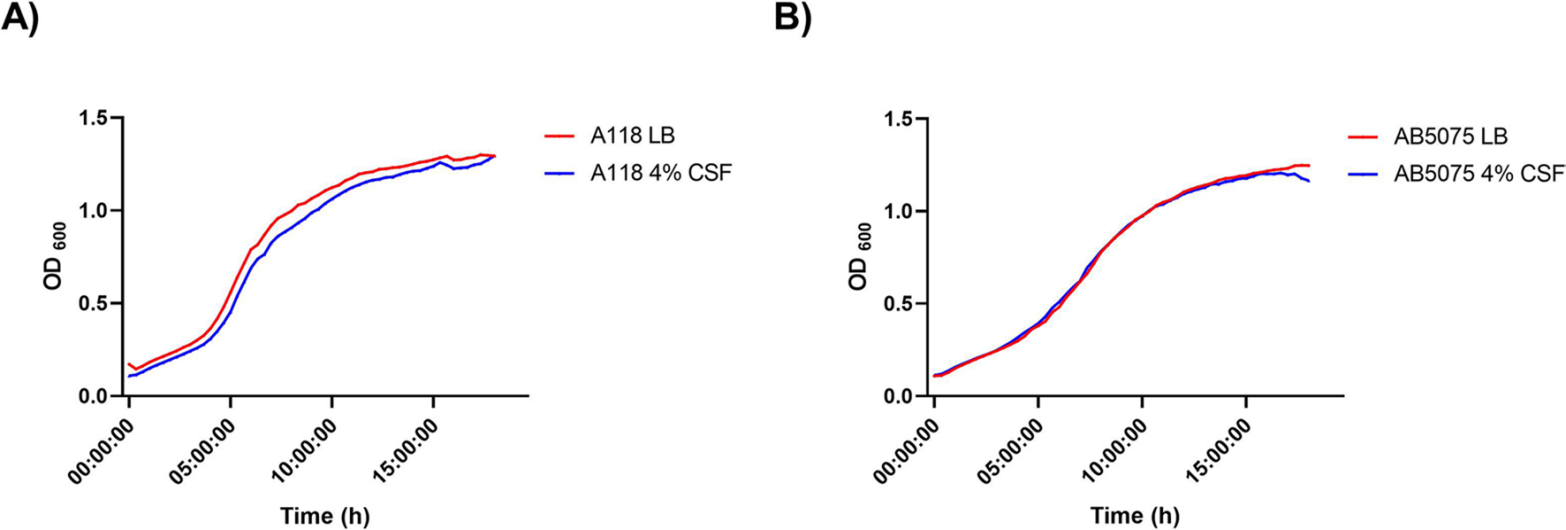
A118 and AB5075 growth curves in LB or LB plus 4% CSF. Strains (**A**) A118 and (**B**) AB5075 were grown in LB or LB supplemented with 4% CSF. Growth curves were conducted in independent experimental triplicates.

The CSF-mediated upregulation of genes coding for the elements necessary for transcription, translation, expression and ATP synthesis was not accompanied by a decrease in generation time (Fig. 1). These results suggest that cells respond to CSF enhancing the expression of pathways that produce specific effects rather than increasing growth capacity.

There are numerous reports supporting the hypothesis that increasing the expression of enzymes involved in transcription, translation, and synthesis of ATP is correlated with faster growth rate^28–32^. However, these growth differences were not evident in either of the *A. baumannii* strains in the presence of CSF. An attractive notion to explain this observation is that the increase in gene expression capabilities is channeled toward the synthesis of cell components necessary for survival in the human body, e.g., adhesins and pilins.

The data described in this set of investigations indicate that certain modifications in the *A. baumannii* metabolism are uncoupled from growth rate. This is not anticipated because bacterial cells are characterized by allocating resources to maximize growth according to the needs for each environmental condition^34^. Our data also suggest that in depleted medium such as CSF, *A. baumannii* may be allocating all possible resources towards metabolism using an uncoupled metabolism to optimize its survival.

### CSF affects the expression of *A. baumannii* virulence genes

We next observed that the addition of CSF to *A. baumannii* A118 cultures induced an increase in the expression of a set of genes that code for virulence-associated functions such as type IV pili, iron uptake systems, the type VI secretion system (T6SS), and poly-N-acetylglucosamine (PNAG) production.

Type IV pili participate in microbial adherence as well as motility (gliding or twitching). While *A. baumannii* lacks flagellum-mediated motility, twitching and surface-associated motility was demonstrated in several strains^35,36^. Numerous studies on twitching and surface-associated motility in *A. baumannii* A118 showed dependence on changes in light and temperature^27^ as well as on the components of the growth media. In particular, addition of HSA resulted in increased motility and concomitantly upregulation of the cognate genes^26^.

**Figure 2:**
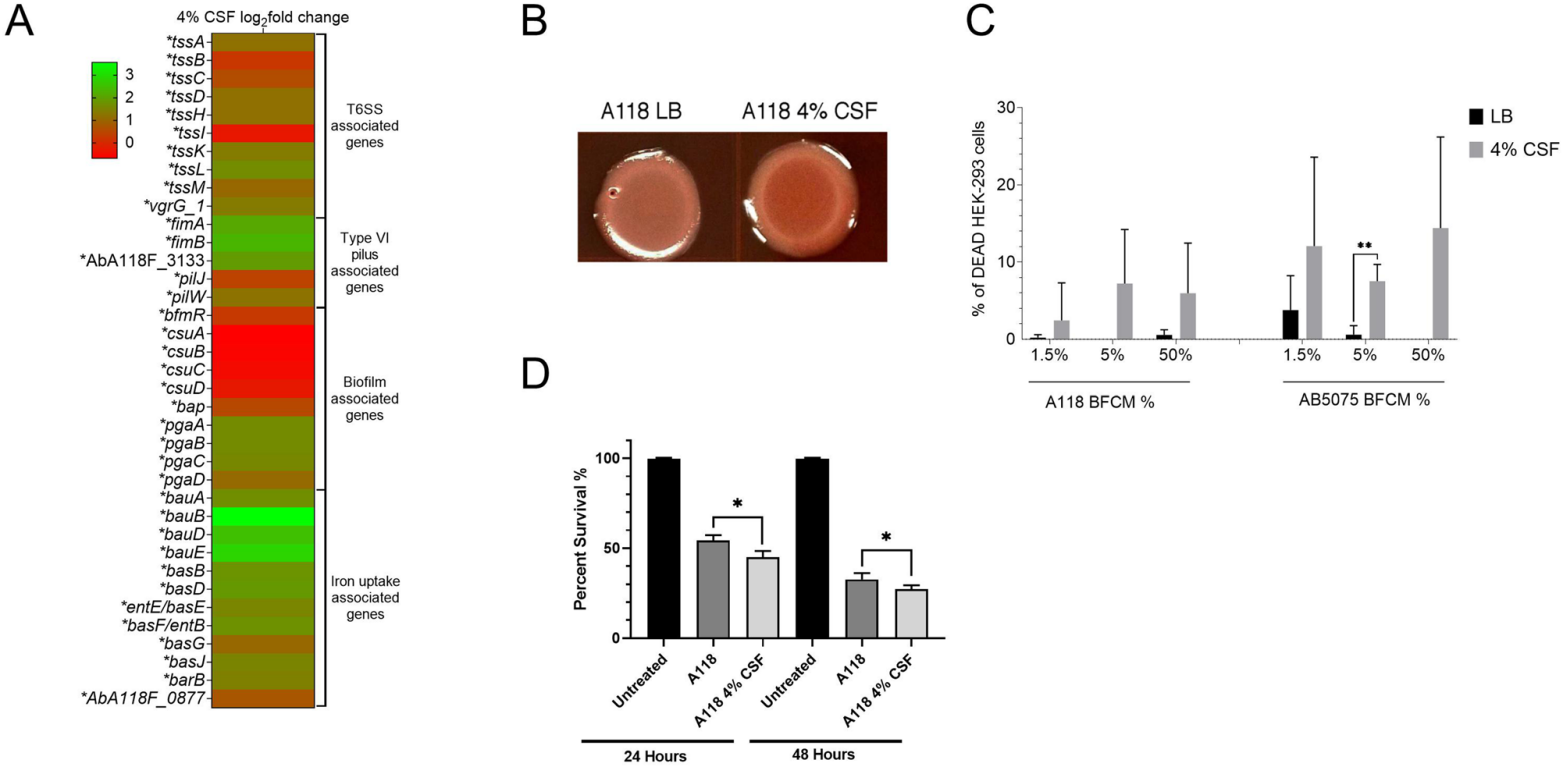
Exposure to CSF can affect multiple virulence factors in *A. baumannii. (A*) Heat map of multiple virulence factor associated genes that were differentially expressed in *A. baumannii* strain A118. Asterisks represent a *P-value* of < 0.05. (**B**) Poly-N-acetylglucosamine (PNAG) assays were conducted with strains A118 in LB or LB supplemented with 4% CSF. (**C**) Percentage viability of HEK-293 cells under exposure to various concentrations of *A. baumannii* strains A118 or AB5075 supplemented with or without 4% CSF. (**D**) Percentage survival of *Galleria mellonella* when inoculated with *A. baumannii* strain A118 with or without 4% CSF.

Exposure of *A. baumannii* A118 to CSF produced an increase in the expression of *pilW* (log_2_fold change 1.22), *pilJ* (log_2_fold change 0*.*43), *fimA* (log_2_fold change 3.09), *fimB* (log_2_fold change 2.32), and the fimbrial protein precursor AbA118F_3133 *(*log_2_fold change 1.91) (Fig. 2A). All of the type IV fimbriae genes have been experimentally shown to be associated with motility, cell adhesion, and biofilm formation^37,38^. In addition, our transcriptomic data showed reduced expression in the biofilm associated genes *csuABCD*, the two-component system response regulator *bfmR,* and the *bap* ortholog (biofilm-associated protein, see Supplementary Table S1). Significant differences were not observed in biofilm formation or motility in the presence of CSF (Fig. S1). This result is in contrast to our previous studies with 4% pleural fluid. The absence of changes in biofilm formation and motility can be explained by the different compositions of each fluid. Pleural fluid is clearly inducing more changes than CSF when comparing the number of genes affected by both fluids (1120 vs 275, respectively). Components such as neutrophils, lymphocytes, monocytes, proteins, reactive oxygen species, and neutralization agents are found in pleural fluid and could contribute to the different effect^22^.

The presence of CSF was also correlated with higher expression of twelve genes associated with the acinetobactin iron uptake system (Fig. 2A and Table S1). These genes are part of the ferric-acinetobactin receptor-translocation machinery (*bauABDE, bauA* log_2_fold change 1.67), the acinetobactin biosynthesis (*basBDFGJ, basD* log_2_fold change 1.86*)* and export (*barB)*^39,40^ (Fig. 2A and Table S1). Besides their direct role in iron uptake in the iron starvation conditions found in the human host, the products of *basD* and *basA* are needed for *A. baumannii* to persist and cause apoptosis of human alveolar epithelial cells^41^. Bacterial iron uptake systems that are virulence factors are usually highly regulated and are induced under conditions of iron starvation. The transcriptomic data showed that, genes that code for functions in siderophore biosynthesis, export, and import are upregulated of in the presence of CSF. This finding adds another regulatory signal that enhances expression of acinetobactin iron uptake system. This increase in expression could be directly related to growth in the host or to biofilm formation, which is dependent on efficient iron uptake^42^.

The structures of bacterial biofilms are usually dependent on polysaccharides such as poly-β-1,6-*N*-acetyl-d-glucosamine (PGA) or cellulose. Previous studies showed that functional production of PGA in *Escherichia coli* depends on the products of four genes, *pgaABCD. pgaC* and *pgaD* are essential for biosynthesis, and *pgaB*, which specifies a *N*-deacetylase, together with *pgaA* are needed to export the polysaccharide from the periplasm to the extracellular milieu^43^. All four homologs were significantly upregulated when *A. baumannii* A118 was cultured in the presence of CSF (log_2_fold change of 1.63, 1.63, 1.56 and 1.08 for *pgaA, pgaB*, *pgaC* and *pgaD*, respectively) (Fig. 2A and Table S1). As expected, Congo red staining showed that *A. baumannii* A118 cells cultured in the presence of CSF produced higher levels of PGA (Fig. 2B).

Another system known to be involved in *A. baumannii’s* virulence is the T6SS^44^. The transcriptomic data showed that nine out of the 14 *A. baumannii* A118 T6SS genes were significantly upregulated in the presence of CSF. These genes *tssABCDHIKLM*, code for essential components of the T6SS (Fig. 2A and Table S1).

Other genes of interest related with resistance and pathogenesis of *A. baumannii* were analyzed. We analyzed the genes involved with antibiotic resistance, quorum sensing, osmotic stress, DNA damage, outer membrane vesicle production, and capsule formation. While many were not found to be differentially expressed, a non-statistically significant upregulation of carO*, which is involved in selective uptake of basic amino acids and also found to be related with carbapenem resistance, was observed^45^. A non-statistically decrease in expression of *bla*_OXA-69_, β-lactamase found in A118 genome, was also observed. Only *abaI*, which codifies for the autoinducer synthesis protein^46^, was differentially expressed among the quorum sensing genes, while the rest where downregulated with the exception of *fadD* (See Fig. S2). In addition, we observed that the osmotic stress regulators, *bet* (a high-affinity choline uptake protein) and *betI* (a transcriptional regulator) were differentially upregulated by a log_2_fold change of -1.20 and -1.40, respectively. For genes associated with capsule formation (K-locus), *pgm* which encodes a phosphomannomutase was upregulated by 1.08 log_2_fold (*P*-value = 2.16 E-21). Regarding the SOS response associated genes, any of them was differentially expressed (See Fig. S2. This result differs from our previous work with pleural fluid exposure, where we found an overrepresentation of the expression of SOS response associated genes difference that can be explain by the presence of reactive oxygen species in pleural fluid.

Lastly, among the genes related with outer-membrane vesicle (OMV) production, *ompA* was the only differentially expressed (Fig. S2). OmpA is known to have a cytotoxic effect and is considered a key virulence factor associated with bacterial biofilm formation, eukaryotic cell infection, resistance to antibiotics and also poses immunomodulatory effects^22,47^.

### CSF enhances the release of *A. baumannii’*s cytotoxic agents

An initial assessment of the effect of CSF on *A. baumannii* virulence was determined using cytotoxicity assays. Bacteria-free conditioned medium (BFCM) obtained from *A. baumannii* A118 and AB5075 cultured in LB with or without CSF was added to human embryonic kidney cells (HEK-293), and the cells were inspected after 1 h.

Figure 2C shows that BFCM samples obtained from CSF-containing *A. baumannii* AB5075 cultures were significantly more cytotoxic than BFCM from cultures that lacked CSF. This increase in cytotoxicity was observed at all tested concentrations, 1.5% BFCM (*P*-value = 0.006), 5% BFCM (*P*-value = 0.001), and 50% BFCM (*P*-value < 0.0001). Conversely, BFCM obtained from *A. baumannii* A118 cultures containing CSF showed an increased cytotoxic effect only at the highest concentration tested (50%) (*P*-value = 0.002). The results of these assays show that CSF induces the release of one or more cytotoxic substances by *A. baumannii* (Fig. 2C).

### CSF-treatment increases *A. baumannii* virulence

The effect of CSF on *A. baumannii’s* virulence was tested using the *Galleria mellonella* model^41,48^. Infection with *A*. *baumannii* A118 cultured in LB plus 4% CSF resulted in increased mortality compared to the infection with cells cultured in LB (Fig. 2D). These results were consistent with the transcriptional changes in expression of virulence genes observed in vitro.

### HSA contribution in *A. baumannii* pathoadaptation when exposed to CSF

Previous work showed that the presence of pleural fluid (PF) is correlated with modifications of the expression of more than 1100 *A. baumannii* genes including many virulence factors involved in motility, biofilm formation, efflux, T6SS, fibrinolytic activity and capsule genes^22^, and with an increase in cytotoxicity and immune evasion^30^. The experiments shown in previous sections indicate that CSF produces effects similar to those observed with PF such as increase in cytotoxicity and changes in expression of virulence genes. Both fluids, PF and CSF, share as a component, HSA. Thus, we posited that this component may be responsible for the effects produced by both human fluids. To test this hypothesis, we compared levels of expression of the Type 1 fimbrial protein FimA*,* the iron transport protein BauD*,* and the Type IV secretion protein VgrG in *A. baumannii* A118 cells cultured in LB or LB supplemented with either CSF or HSA.

RNA-seq showed that *bauD* and *vgrG*, were up-regulated in both, CSF (log_2_fold-change = 2.5247 and 1.3611 respectively) and HSA (log_2_fold-change = 0.8046 and 1.1063 respectively)^26^. On the other hand, the *fimA* gene was up-regulated with a log_2_ fold change of 3.0938 in the presence of LB supplemented with CSF but surprisingly, it was slightly down-regulated in medium supplemented with HSA (log_2_ fold change of 1.6434) (non-statically significant considering a *P*-value < 0.05)^26^. While the results obtained with *bauD* and *vgrG* seemed to uphold the hypothesis, those produced when assessing expression of *fimA* did not seem to support that HSA is responsible for CSF-induced upregulation.

**Figure 3:**
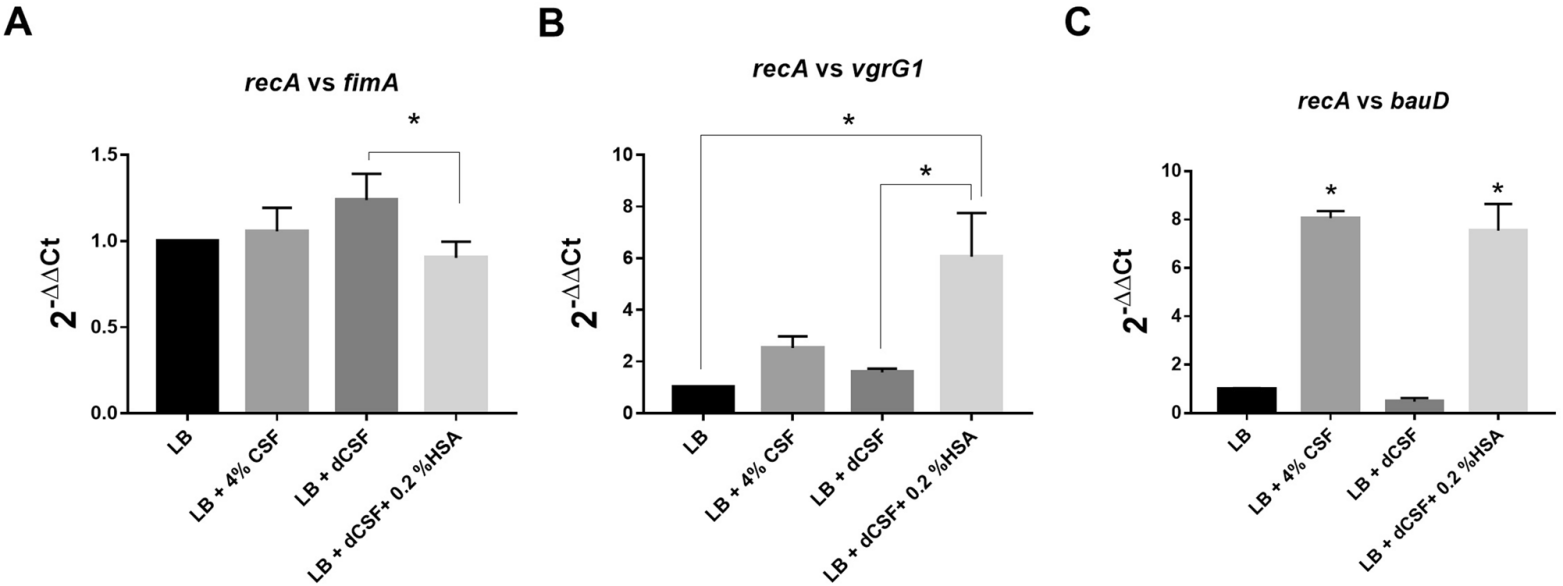
HSA is an essential component for the differential expression of genes in *A. baumannii*. *A. baumannii* A118 cells were cultured in LB or LB supplemented with one of the following: 4% CSF, HSA-depleted CSF (dCSF), or dCSF + 0.2% HSA and its cDNA was synthesized. RT-qPCR was conducted with three genes (**A**) *fimA* encoding gene, (**B**) *vgrG1*, and (**C**) *bauD.* Shown are the means of the results obtained from three independent experiments. The *y* axis refers to the fold difference of each gene to the threshold cycle (C_T_) values corresponding to *recA*; the standard deviation SD is shown. Asterisks indicate significant differences among treatments, as determined by ANOVA followed by Tukey’s multiple comparison test (*P* < 0.05).

To confirm the results obtained by RNA-seq, another set of experiments was carried out measuring levels of expression by quantitative PCR using total RNA from *A. baumannii* A118 cells cultured in LB or LB supplemented with either CSF, HSA-depleted CSF (dCSF), or dCSF + 0.2% HSA (Fig. 3A-C). Expression of *vgrG1* was slightly up-regulated in presence of 4% CSF (2.527-fold; *P*-value 0.4113) and dCSF (1.586-fold; *P*-value 0.9040). However, supplementation of LB with dCSF + 0.2% HSA, resulted in a robust increase of 6.065-fold (*P*-value 0.0152) and 3.825-fold (*P-*value 0.0234) with respect to LB or LB supplemented with dCSF, respectively (Fig. 3B). Expression of *bauD* was increased by 8.064-fold (*P*-value 0.0009) and 7.544-fold (*P*-value 0.0012) in cells growing in LB supplemented with CSF or dCSF + 0.2% HSA, respectively. Moreover, a 15.847-fold increase (*P*-value 0.0009) was observed in levels of expression of *bauD* in cells growing in LB supplemented with dCSF + 0.2% HSA with respect to those in cells growing in LB supplemented with dCSF (Fig. 3C). Conversely, while a slight increase in *fimA* expression was noted in cells growing in LB supplemented with CSF (1.057-fold, *P*-value 0.9190) or dCSF (1.238-fold, *P*-value 0.1349), there was a decrease of 1.107-fold (*P*-value 0.6526) when the medium was LB supplemented with dCSF + 0.2% HSA. A comparison between levels of expression in cells growing in LB supplemented with dCSF + 0.2% HSA or dCSF showed that the presence of HSA resulted in 1.370-fold decrease (*P*-value 0.0384) (Fig. 3A). These results confirmed that while HSA may be responsible for increasing expression of certain genes, it is not for others like *fimA*. In fact, it seems to induce a weak but consistent inhibition of expression of *fimA*. Future studies will need to carry out to determine if other components of CSF produce an increase in *fimA* expression.

Bacterial cells express genes that code for factors that allow growth in the hostile environments they encounter upon invading the human body. The results shown in this section indicate that HSA is one of the signals that triggers the expression of several *A. baumannii* genes when the bacterial cells are in contact with HSA-containing fluids, PF or CSF. Furthermore, our previous studies showed that HSA also enhances DNA acquisition through modulation of natural competence-related gene expression and affects the expression of genes related to motility, efflux pumps, pathogenicity and antibiotic resistance, among others^23,26^. These characteristics are not unique to *A. baumannii*, various bacterial pathogens and protozoa^49–52^ modify gene expression to adapt and thrive within the host utilizing HSA as one of the signals. For example, in *Bordetella pertussis*, the causative agent of the whooping cough, albumin combined with calcium induces an increase in production and release of the major toxin, adenylate cyclase toxin (ACT)^52^. Another example is the case of *Pseudomonas aeruginosa*, in which the presence of albumin is correlated with increased expression of iron-controlled genes (*pvdS* and *regA*)^53^. In summary, our observations, together with the evidence available from studies with other bacteria, suggest an important role of HSA as signal for expression of genes and systems essential for survival within the human body.

## Conclusion

**Figure 4:**
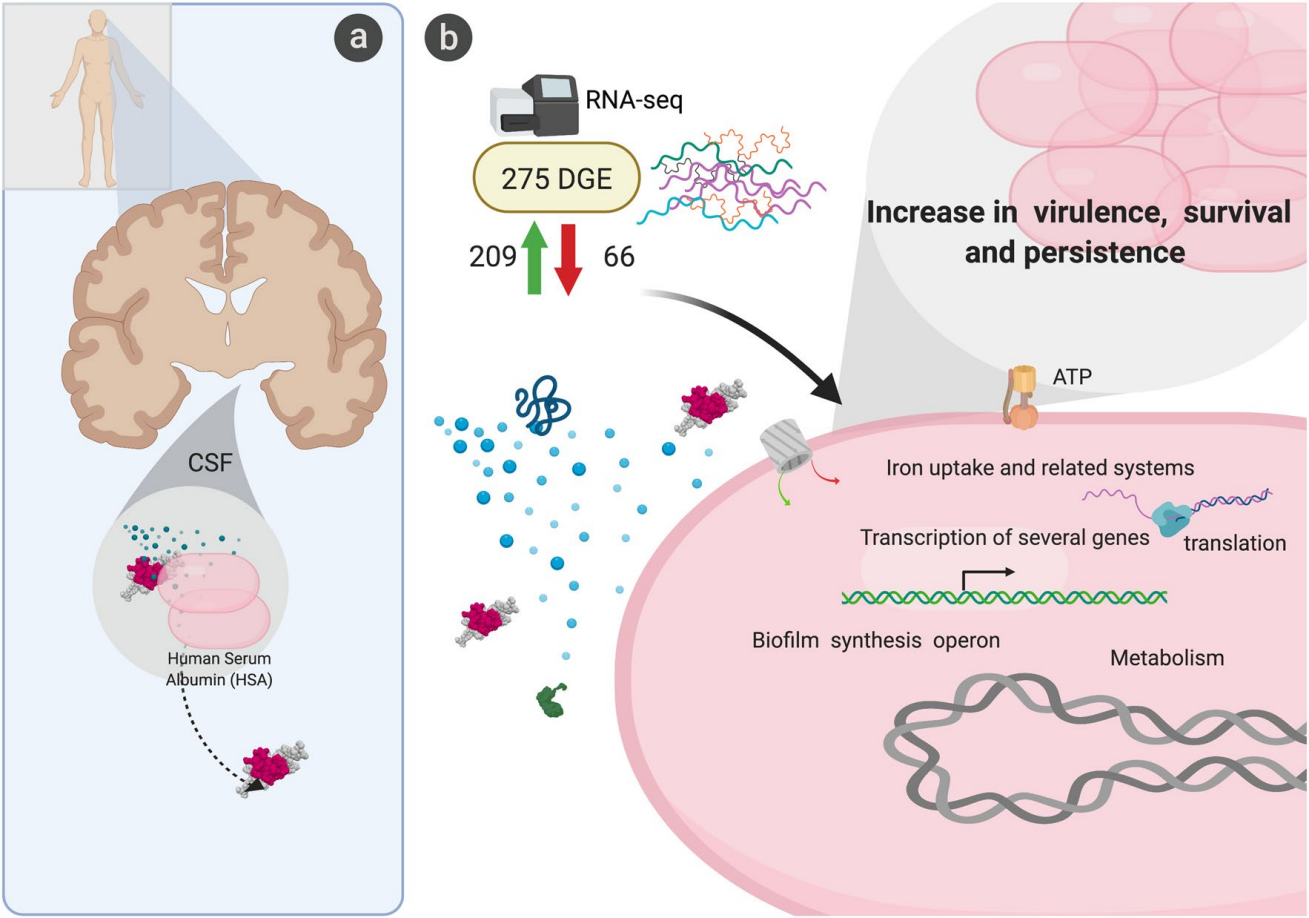
Graphical representation of *A. baumannii’s* transcriptomic and behavioral response to CSF. (**A**) General representation showing possible association of colonized *A. baumannii* in CSF towards HSA. (**B**) Hypothetical schematic showing possible role of HSA in inducing differential gene expression in *A. baumannii.* The differential expression of genes associated with metabolism, iron uptake systems, and translation machinery, among other, were found in the 275 DEGs. Created with BioRender.com.

*A. baumannii* is one of many causative agents of nosocomial bacterial meningitis, an infection associated with high morbidity and mortality rates. During the infection, bacteria can be found in CSF. In fact, of the several methods available for diagnosis of bacterial meningitis, CSF culture is the most favored^54^. This study describes changes in expression of numerous genes when *A. baumannii* is exposed to CSF (Fig. 4). These genes code for a variety of proteins that participate in the gene expression machinery, energy production, motility, metabolism, survival, and virulence factors among others. Our results in combination with previous work suggest that HSA may be a contributor in signaling these transcriptomic responses (Fig. 4). HSA is one of the main components of CSF and is also present in blood and PF, all body fluids that trigger similar responses in bacteria. Utilizing HSA as the signal for gene expression of elements that facilitate progression of the infection is an intelligent strategy that permits bacteria to sense the presence of human environments. However, not all strains respond equally when HSA is present, slight differences were identified when comparing *A. baumannii* strains A118 and AB5075. These changes are correlated with differences in levels of pathogenicity and probably the kind of infections that are more commonly caused by each variant.

Another remarkable effect of HSA on *A. baumannii* is the augmentation of natural competency^26,55^. Traglia et *al.* proposed that a random coil stretch in the structure of HSA is responsible for increasing the ability of *A. baumannii* to take up DNA. Identifying factors that are similar between human host environments may be potential therapeutic target candidates. Considering the pleiotropic effects caused by the presence of HSA on *A. baumannii*, an alternative path to design therapeutic agents against this infection could be to identify compounds that interfere with the ability of *A. baumannii* to detect HSA. Compounds that interact with the HSA regions that are detected by *A. baumannii* could mask the presence of the protein impeding expression of the necessary systems for survival and progression of the infection.

## Material and methods

### Bacterial strains and human fluids

Two *A. baumannii* strains already used in previous studies^27–29^, exhibiting different degree of susceptibility and virulence were used. *A. baumannii* A118 strain is known to be susceptible to variety of antibiotics^28,56^ and *A. baumannii* AB5075 possesses increased virulence and is resistant to many antibiotics^29^.

The Pooled Human Cerebrospinal Fluid (CSF) sample was acquired from Innovative Research, MI, which is a certified vendor that obtains human samples from Food and Drug Administration (FDA)-approved facilities. The samples were collected from normal healthy individual donors and pooled. Lysogeny Broth, LB, supplemented with 4% CSF was used for all CSF conditions. The following concentration was used since 4% of other human fluids has been used in previously studies^22,30,57^.

### RNA extraction and sequencing

*A. baumannii* colonies (A118 and AB5075) were suspended in LB with or without 4% CSF and incubated with agitation for 18 h at 37 °C. Overnight cultures were then diluted 1:10 in fresh LB broth and incubated with agitation for 7 h at 37 °C. RNA was extracted from each strain using the TRI REAGENT Kit (Molecular Research Center, Inc., Cincinnati, Ohio, USA) as previously described^26^. Total RNA extractions were performed in two biological replicates for each condition.

RNA sequencing was outsourced to Novogene (Novogene Corporation, CA) for mRNA-seq analysis, which includes rRNA depletion, library preparation following the protocols of the NEBNext Ultra II Directional RNA Library Prep Kit for Illumina (New England Biolabs) (New England Biolabs) and HiSeq 2500 paired-end 150 bp sequencing**.**

### RNA–seq data analysis

RNA-seq reads (GEO accession No GSE153967) corresponding to *A. baumannii* strain A118 and AB5075 exposed to LB or LB plus 4% CSF were analyzed as follows. Trimming of low-quality bases at the ends of the reads to a minimum length of 100 bp and removal of Illumina adaptor sequences was performed using Trimmomatic^58^. FastQC (www.bioinformatics.babraham.ac.uk/projects/fastqc/) was used to assess the quality of the reads before and after trimming. Burrows-Wheeler Alignment software (BWA) was used to align the RNA-seq reads to sequences of the whole genome shotgun sequencing project of strain *Acinetobacter baumannii* A118F (DDBJ/ENA/GenBank accession VCCO01000000). FeatureCounts was used to calculate the read counts per gene, and differential expression analysis was performed using DEseq2^59,60^. Principal component analysis (PCA) and gene expression heat map with clustering dendrograms of the RNA-seq data analysis of LB and CSF treatments are shown in Supplementary Fig. S3. Features exhibiting FDR < 0.05 and log_2_fold change > 1 were considered statistically significant.

### Gene ontology (GO) analysis

GO terms were retrieved from UniProt for the best BLASTx hits to *A. baumannii* A118F genes. Using GO.db Bioconductor annotation data package in R language, GO terms and ancestor terms were assigned for all DEGs from this study. GO enrichment analysis was performed using custom-made scripts as described previously^61^. The enrichment factor was estimated as the ratio between the proportions of genes associated with a particular GO category present in the dataset under analysis, relative to the proportion of the number of genes in this category in the whole genome. *P*-values were calculated using the Fisher Exact Test and adjusted by the Benjamini–Hochberg method.

### Growth curves

Growth curves of both strains, A118 and AB5075, were conducted on 96-well plates. Overnight cultures were subcultured 1:50 in LB or LB + 4% CSF and incubated for 18 h at 37 °C with medium shaking. OD_600_ nm was measured every 20 min using a Synergy 2 multi-mode plate reader (BioTek, Winooski, VT, USA) and Gen5 microplate reader software (BioTek). To study the effect of different carbon sources, both strains were culture overnight under different condition (LB broth and LB broth + 4% CSF).

### Motility and biofilm assays

Motility and biofilms assays were performed as previously described^26^. A118 and AB5075 cells were cultured in LB broth with or without 4% CSF. Bacterial cells were incubated with agitation for 18 h at 37 °C. Experiments were performed in triplicate, with at least three technical replicates per biological replicate.

### Cytotoxicity assays

In a Nunclon Delta Surface opaque 96-well microplate (ThermoScientific), we added colorless DMEM, 4% CSF, and A118 or AB5075 BFCM diluted in LB broth to make 50 μL of BFCM at final concentrations of 1.5%, 5%, and 50%. An additional 50 μL of ATCC HEK-293 cells at a concentration of 1 × 10^6^ cells/mL in colorless DMEM were suspended in the well and intoxicated for 1 h at 37 °C, 5% CO_2_. CellTiter-Glo Reagent (100 μl) was added to each experimental and standard curve well and then placed on an orbital shaker for 2 min. Following mixing, plates were incubated at room temperature for 10 min to stabilize the luminescent signal. The viability of HEK cells was measured at room temperature using the “all” luminescence function of SpectraMax M3.

### *Galleria mellonella* infection model

To assess the virulence of *A*. *baumannii* with and without CSF in vivo, the *G*. *mellonella* insect model of infection was used^62^. Larvae weighing between 200 and 400 mg were maintained on wood chips in the dark at 4 °C. *A*. *baumannii* A118 was grown overnight in either LB or LB with 4% CSF. An equivalent of 1.0 OD_600_ unit of overnight culture was pelleted and resuspended in 1 mL of cold sterile 20 mM phosphate buffered saline, pH 7.4 (PBS). The cells were further diluted 1:10 in sterile PBS and used for injections. A Hamilton syringe was used to inject 5 μL of the diluted bacterial suspension via the left proleg of each larva. A control group of untreated larvae was used to assess overall larval viability for the duration of the assay. One hundred *G*. *mellonella* larvae were used in each condition and incubated at (37 °C) in a sterile Petri dish for 24 h intervals for 48 h total. Larvae viability was monitored by observing response to gentle prodding with a glass rod; those with no response were considered dead. Four replicates with 100 larvae per Petri dish were performed for each condition.

### PNAG assays

To study extracellular matrix (ECM) production, microcolony biofilm was used as model system. 5 μl of overnight cultures of A118 cultured in LB broth and LB broth + 4% CSF were inoculated on LB agar and supplemented with Congo red as previously described^63^. Plates were incubated at 28 °C in static incubator for up to 48hs. Results were recorded at 24 h with a Plugable USB 2.0 Digital Microscope.

### HSA depletion

HSA was depleted from CSF by placing 1 mL of CSF into a 30 kDa Amicon Ultra Centrifugal Filter (Millipore, Temecula, CA, United States) and the solution was centrifuged at 20,000 × g for 10 min. To identify HSA was successfully depleted, an SDS-PAGE was conducted that contained 4% CSF, depleted CSF (dCSF), and dCSF plus 0.2% HSA (Fig. S4).

### RT-qPCR

Previously extracted and DNase-treated RNA from *A. baumannii* strain A118 grown in LB, 4% CSF, 4% depleted CSF and 4% depleted CSF + 0.2% HSA, were synthesized to cDNA using the manufacturer protocol provided within the iScript Reverse Transcription Supermix for qPCR (Bio-Rad, Hercules, CA, United States). The cDNA concentrations were measured with a DeNovix DS-11 + spectrophotometer; each sample was then diluted to a concentration of 50 ng/μl. qPCR was conducted using the iQ SYBR Green Supermix through the manufacturer’s instructions. At least three biological replicates of cDNA were used and were run in quadruplet. All samples were then run on the CFX96 Touch Real-Time PCR Detection System (Bio-Rad, Hercules, CA, United States).

The transcript levels of each sample were normalized to the *recA* rRNA transcript levels for each cDNA sample. The relative quantification of gene expression was performed using the comparative threshold method 2^−ΔΔCt^. The ratios obtained after normalization were expressed as folds of change compared with cDNA samples isolated from bacteria cultures on LB. Asterisks indicate significant differences as determined by ANOVA followed by Tukey’s multiple comparison test (*P* < 0.05), using GraphPad Prism (GraphPad software, San Diego, CA, United States).

### Statistical analysis

All experiments were performed at least in technical and biological triplicate. Data was expressed as means ± standard deviation. Statistical analysis using Mann–Whitney test or ANOVA followed by Tukey’s multiple comparison test were performed using GraphPad Prism (GraphPad software, San Diego, CA, USA), and a *P*-value < 0.05 was considered statistically significant.

All procedures performed in this study were in accordance with the CSUF Institutional Biosafety Committee Approval plan (DBH117-01) and are in compliance with the NIH, CDC, OSHA and other environmental and occupational regulations.

## Supplementary information

Supplementary figures

Supplementary Tables
