# Supplementary material for: Cerebrospinal fluid (CSF) augments metabolism and virulence expression factors in Acinetobacter baumannii
Source: Sci Rep. 2021 Feb 26;11:4737. doi: 10.1038/s41598-021-81714-6 (PMC7910304; doi:10.1038/s41598-021-81714-6)

Cerebrospinal fluid (CSF) augments metabolism and virulence expression factors in *Acinetobacter baumannii*

Jasmine Martinez[1], Chelsea Razo-Gutierrez[1], Casin Le[1], Robert Courville[1], Camila Pimentel[1], Christine Liu[1], Sammie E. Fuang[1], Marisel R. Tuttobene[1], Kimberly Phan[1], Alejandro J. Vila[2,3], Parvin Shahrestani[1], Veronica Jimenez[1], Marcelo E. Tolmasky[1], Scott A. Becka[4], Krisztina M. Papp-Wallace[4,5,6], Robert A. Bonomo[4,5,6], Alfonso Soler-Bistue[7], Rodrigo Sieira[8], Maria Soledad Ramirez[1*].

[1]Center for Applied Biotechnology Studies, Department of Biological Science, College of Natural Sciences and Mathematics, California State University Fullerton, Fullerton, California, USA,

[2]Instituto de Biología Molecular y Celular de Rosario (IBR, CONICET-UNR), Rosario, Argentina,

[3]Área Biofísica, Facultad de Ciencias Bioquímicas y Farmacéuticas, Universidad Nacional de Rosario, Rosario, Argentina

[4]Research Service and GRECC, Louis Stokes Cleveland Department of Veterans Affairs Medical Center, Cleveland, Ohio, USA,

[5]Departments of Medicine, Pharmacology, Molecular Biology and Microbiology, Biochemistry, Proteomics and Bioinformatics, Case Western Reserve University School of Medicine, Cleveland, Ohio, USA,

[6]CWRU-Cleveland VAMC Center for Antimicrobial Resistance and Epidemiology (Case VA CARES), Cleveland, Ohio, USA,

[7]Instituto de Investigaciones Biotecnológicas, Universidad Nacional de San Martín-Consejo Nacional de Investigaciones Científicas y Técnicas, San Martín, Buenos Aires, [8]Fundación Instituto Leloir – IIBBA CONICET, Buenos Aires, Argentina.

Running Title: CSF boosts *A. baumannii* survival

Keywords: *Acinetobacter baumannii*, CSF, metabolism, virulence, growth, transcription, survival.

***Corresponding author.**

María Soledad Ramírez, PhD.

Assistant Professor

Dept. Biological Science

California State University Fullerton

800 N State College Blvd

Fullerton, CA 92831

e-mail: msramirez@fullerton.edu

Tel: +1 657-278-4562

Supplementary material

Figure S1. Effect of CSF exposure towards *A. baumannii* surface associated motility and biofilm formation. A) Surface associated motility assays were conducted with strains A118 and AB5075 with or without exposure to 4% CSF. B) Biofilm formation assays were conducted with strains A118 and AB5075 with or without exposure to 4% CSF. Values are represented as OD_{580}/OD_{600}.

FIGURE S2. RNA-seq data analysis of *A. baumannii* genes associated with other virulence and stress response factors. RNA-seq data was organized into heat maps showing *A. baumannii* genes associated with A) Quorum sensing/ quenching, B) SOS response, C) antibiotic resistance, D) osmotic stress, E) K-locus, and outer membrane vesicle production. Asterisks represent genes that were differentially expressed.

FIGURE S3. Bioinformatic analysis of RNA-seq data from LB- or 4% CSF-treated *A. baumannii* A118. A) PCA plot of all RNA-seq samples. Biological replicates of the same treatment are indicated by color in the legend. B) Heat map of the expression profiles of the 200 genes displaying the highest variance across samples based on DESeq2 analysis of read count data. Sample-wise (columns) clustering dendrogram is shown.

FIGURE S4. SDS-page of CSF, dCSF, dCSF plus HSA and HSA. A) 10 ul of CSF, dCSF, dCSF plus HSA and HSA, B) 1/10 and 1/100 dilutions of CSF, dCSF, dCSF plus HSA and HSA.

FIGURE S1

A

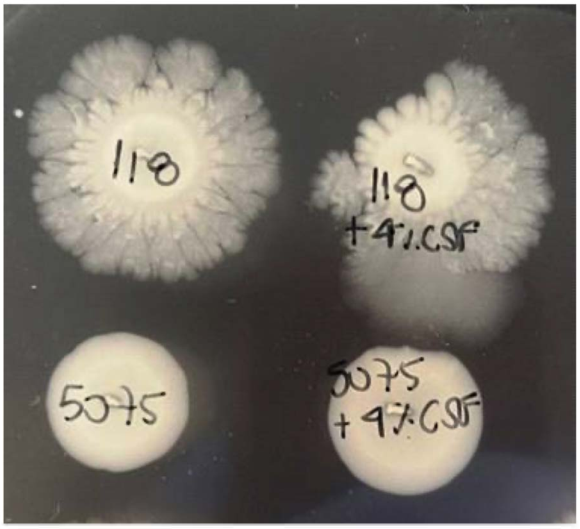

118
118
+4%CSF
5075
5075
+4%CSF

B

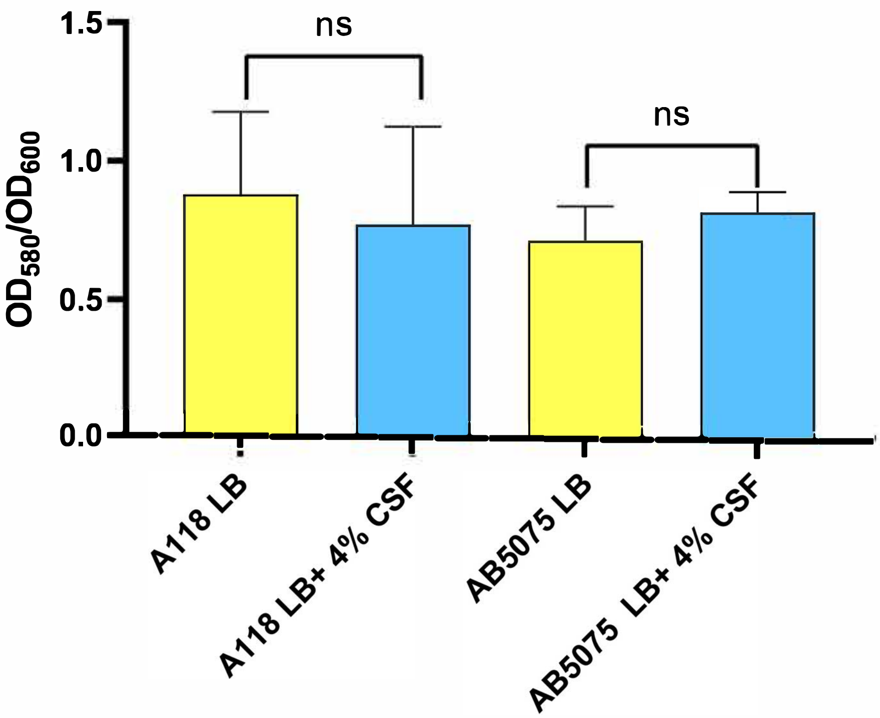

1.5
1.0
0.5
0.0
OD580/OD600
ns
ns
A118 LB
A118 LB+ 4% CSF
AB5075 LB
AB5075 LB+ 4% CSF

FIGURE S2

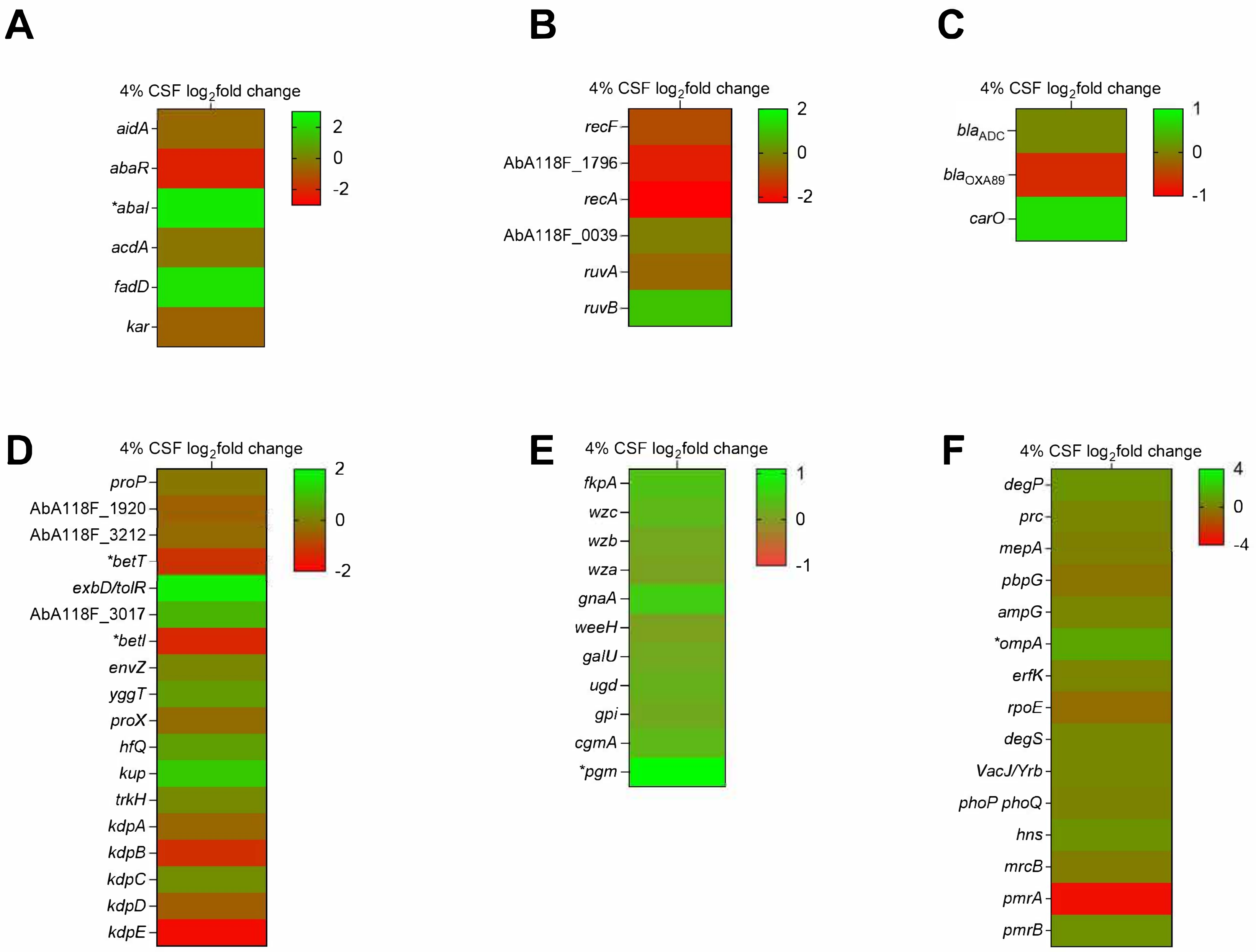
A
4% CSF log2fold change
aidA
abaR
*abaI
acdA
fadD
kar
2
0
-2
B
4% CSF log2fold change
recF
AbA118F_1796
recA
AbA118F_0039
ruvA
ruvB
2
0
-2
C
4% CSF log2fold change
blaADC
blaOXA89
carO
1
0
-1
D
4% CSF log2fold change
proP
AbA118F_1920
AbA118F_3212
*betT
exbD/tolR
AbA118F_3017
*betI
envZ
yggT
proX
hfQ
kup
trkH
kdpA
kdpB
kdpC
kdpD
kdpE
2
0
-2
E
4% CSF log2fold change
fkpA
wzc
wzb
wza
gnaA
weeH
galU
ugd
gpi
cgmA
*pgm
1
0
-1
F
4% CSF log2fold change
degP
prc
mepA
pbpG
ampG
*ompA
erfK
rpoE
degS
VacJ/Yrb
phoP phoQ
hns
mrcB
pmrA
pmrB
4
0
-4

FIGURE S3

A

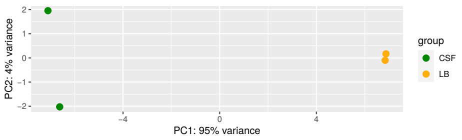
PC2: 4% variance
2
1
0
-1
-2
-4
0
4
PC1: 95% variance
group
CSF
LB

B

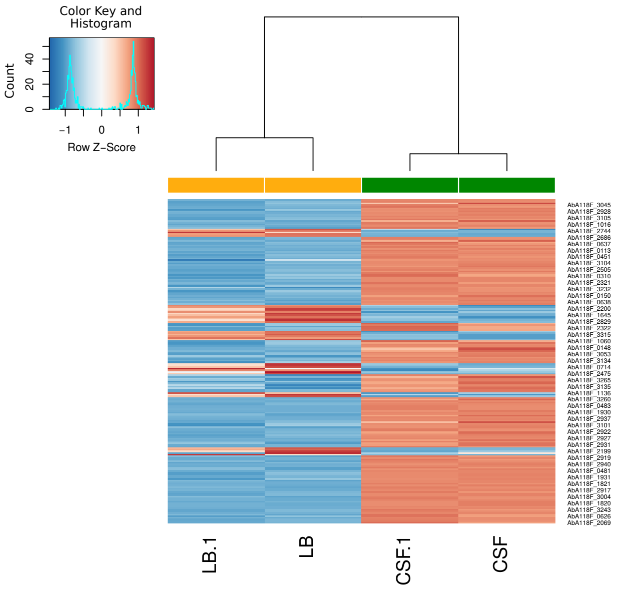
Color Key and Histogram
Count
0
20
40
-1
0
1
Row Z-Score
AbA118F_3045
AbA118F_2928
AbA118F_3105
AbA118F_1016
AbA118F_2744
AbA118F_2686
AbA118F_0637
AbA118F_0113
AbA118F_0451
AbA118F_3104
AbA118F_2505
AbA118F_0310
AbA118F_2321
AbA118F_3232
AbA118F_0150
AbA118F_0638
AbA118F_2200
AbA118F_1645
AbA118F_2829
AbA118F_2322
AbA118F_3315
AbA118F_1060
AbA118F_0148
AbA118F_3053
AbA118F_3134
AbA118F_0714
AbA118F_2475
AbA118F_3265
AbA118F_3135
AbA118F_1136
AbA118F_3260
AbA118F_0483
AbA118F_1930
AbA118F_2937
AbA118F_3101
AbA118F_2922
AbA118F_2927
AbA118F_2931
AbA118F_2199
AbA118F_2919
AbA118F_2940
AbA118F_0481
AbA118F_1931
AbA118F_1821
AbA118F_2917
AbA118F_3004
AbA118F_1820
AbA118F_3243
AbA118F_0626
AbA118F_2069
LB.1
LB
CSF.1
CSF

FIGURE S4

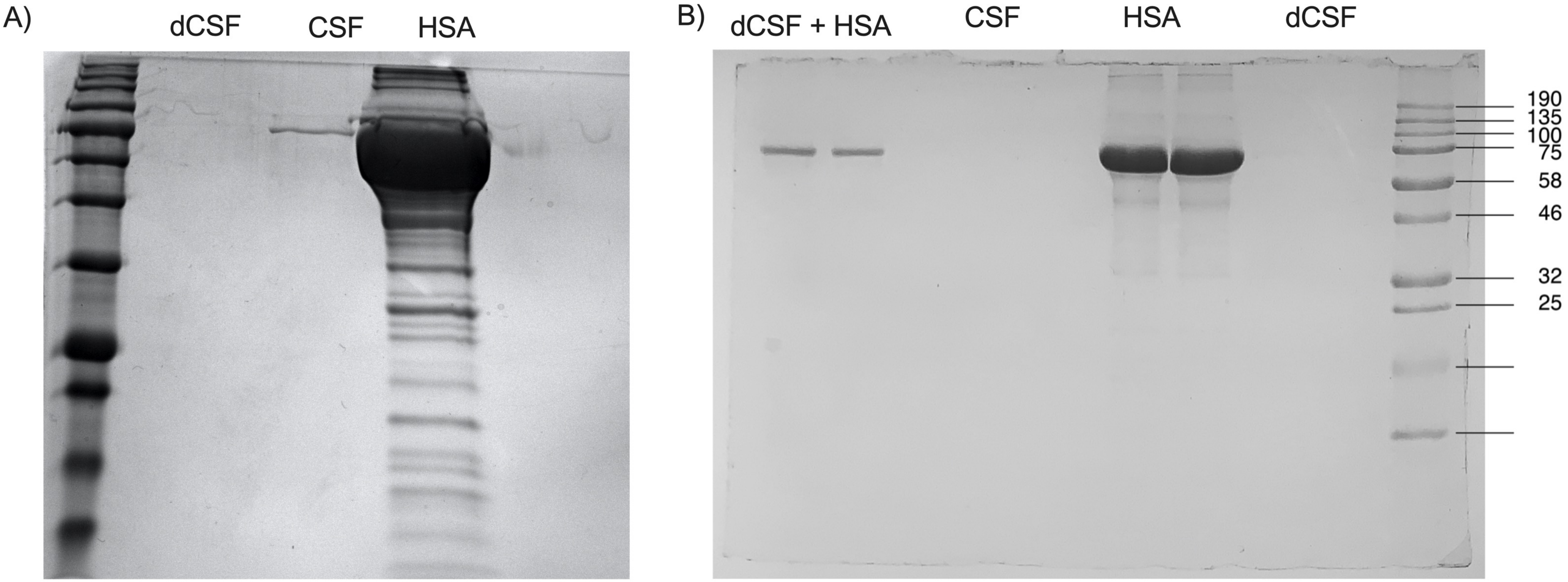

Supplement: Supplementary file 1 — Supplementary figures [file 41598_2021_81714_MOESM1_ESM.pdf]
